# Integrated exercise program in opioid agonist therapy clinics and effect on psychological distress: study protocol for a randomized controlled trial (BAReAktiv)

**DOI:** 10.1186/s13063-024-07993-2

**Published:** 2024-02-29

**Authors:** Einar Furulund, Tesfaye Madebo, Karl Trygve Druckrey-Fiskaaen, Jørn Henrik Vold, Mette Hegland Nordbotn, Eivin Dahl, Sindre M. Dyrstad, Torgeir Gilje Lid, Lars T. Fadnes, for the ATLAS4LAR study group, Vibeke Bråthen Buljovcic, Jan Tore Daltveit, Trude Fondenes, Per Gundersen, Beate Haga Trettenes, Siv-Elin Leirvåg Carlsen, Maria Olsvold, Marianne Cook Pierron, Christine Sundal, Maren Borsheim Bergsaker, Tone Lise Eielsen, Torhild Fiskå, Marianne Larssen, Eirik Holder, Ewa Joanna Wilk, Mari Thoresen Soot

**Affiliations:** 1 Centre for Alcohol and Drug Research, Stavanger University Hospital, Stavanger, Norway; 2 Department of Addiction Medicine, Bergen Addiction Research, Haukeland University Hospital, Bergen, Norway; 3 Department of Global Public Health and Primary Care, University of Bergen, Bergen, Norway; 4 Department of Respiratory Medicine, Stavanger University Hospital, Stavanger, Norway; 5 Department of Addiction, Stavanger University Hospital, Stavanger, Norway; 6 Department of Public Health, University of Stavanger, Stavanger, Norway; 7 Division of Psychiatry, Haukeland University Hospital, Bergen, Norway; 8 Department of Education and Sport Science, University of Stavanger, Stavanger, Norway

**Keywords:** Opiate substitution treatment, Motor activity, Exercise therapy, Substance-related disorders, Intervention, Outpatient, Behavioral intervention

## Abstract

**Background:**

Substance use disorder is associated with unhealthy lifestyle choices, resulting in adverse social and health consequences. People with opioid use disorder receiving opioid agonist therapy, in particular, have high morbidity and reduced quality of life. Physical activity is recommended as an adjunctive treatment for people with substance use disorder, but there is minimal evidence from randomized controlled trials on the effects of this among people with substance use disorder receiving opioid agonist therapy.

**Methods:**

BAReAktiv is a multicentre randomized controlled trial. The study aims to recruit 324 patients receiving opioid agonist therapy (parallel groups randomized 1:1 to integrated exercise intervention or control, superiority trial). A 16-week group-based integrated exercise intervention with workouts twice a week. The exercise program consists of endurance and resistance training. The target group will be patients 18 years and older receiving opioid agonist therapy in outpatient clinics in several centers in Western Norway. The primary outcome of the study is the effect on psychological distress measured by Hopkins’ symptom checklist with ten items. Secondary outcome measures include physical functioning assessed with a 4-min step test, activity level, fatigue symptoms, quality of life, and changes in inflammation markers. This study will provide improved knowledge on the effects of an integrated exercise program in opioid agonist therapy.

**Discussion:**

Systematically integrating exercise programs for people receiving opioid agonist therapy could lead to a shift towards a stronger focus on health behaviors in outpatient care. Integrating exercise could benefit patient recovery and reduce disease burden. Further scale-up will be considered if the provided exercise program is safe and effective.

**Trial registration:**

ClinicalTrials.gov. NCT05242848. Registered on February 16, 2022.

## Administrative information

Note: The numbers in curly brackets in the protocol refer to SPIRIT checklist item numbers. The order of the items has been modified to group similar items (see: http://www.equator-network.org/reporting-guidelines/spirit-2013-statement-defining-standard-protocol-items-for-clinical-trials/).

**Table:** 

Title {1}	Integrated exercise program in opioid agonist therapy clinics and effect on psychological distress: study protocol for a randomized controlled trial (BAReAktiv)
Trial registration {2a–2b}	ClinicalTrials.gov.no NCT05242848. Date of registry February 16, 2022
Protocol version {3}	Version 3, 04 October 2023
Funding {4}	The study was funded by Western Norway Regional Health Authority («Strategiske forskningsmidler» through the ATLAS4LAR-project) with Department of Addiction Medicine, Haukeland University Hospital as responsible institution
Author details {5a}	^1^ Centre for Alcohol and Drug Research, Stavanger University Hospital, Stavanger, Norway; ^2^ Bergen Addiction Research, Department of Addiction Medicine, Haukeland University Hospital, Bergen, Norway; ^3^ Department of Global Public Health and Primary Care University of Bergen, Bergen, Norway; ^4^ Department of Respiratory Medicine, Stavanger University Hospital, Stavanger, Norway; ^5^ Department of addiction, Stavanger University Hospital, Stavanger, Norway; ^6^ Department of Public Health, University of Stavanger, Stavanger, Norway ^7^ Division of Psychiatry, Haukeland University Hospital, Bergen, Norway ^8^ Department of Education and Sport Science, University of Stavanger, Stavanger, Norway ^9^ List of Members of the ATLAS4LAR Study Group (see acknowledgment)
Name of contact information for the trial sponsor {5b}	Western Norway Regional Health Authority, Postboks 303 Forus, 4066 Stavanger, Norway
Role of sponsor {5c}	Funders did not participate in study design, and will not be involved in data collection and analysis, decision to publish, or preparation of the manuscript

###  Background and rationale {6a}

Substance use disorders are associated with unhealthy lifestyle choices, resulting in adverse social and health consequences [1, 2]. People with opioid use disorder have particularly high morbidity, reduced physical fitness and quality of life [3–5], and a higher risk of premature death [6, 7].

Physical activity is defined as any bodily movement produced by skeletal muscles, which requires energy expenditure. Exercise is planned, structured, repetitive, and intentional physical activity intended to improve or maintain physical fitness [8]. Exercise has been suggested as an adjunctive treatment strategy for people with substance use disorder [7], due to its positive effects on health, quality of life, and reducing the burden of substance use disorder patterns [9, 10]. People with opioid dependence receiving opioid agonist therapy (OAT) often have significant mental health problems and calls for improved mental and physical health assessment and patient care [11, 12]. Physical activity and exercise can be effective adjunctive treatment methods for reducing nicotine, alcohol, and drug use [7, 13, 14]. Still, we need more knowledge about the effects of exercise as part of OAT, and how to best integrate an exercise program into OAT [15]. Few studies have investigated the effects of an exercise program on OAT patients [16–21], and none of these are randomized controlled trials with power to assess effects. Results from these studies suggest that exercise interventions could be desirable on physical fitness [19], substance use [16], and quality of life [19]. However, the potential effect is uncertain. Further research on exercise intervention would therefore be beneficial [12].

### Objectives {7}

This paper describes the BAReAktiv protocol. The primary objective is to evaluate the effect of a 16-week exercise intervention on the level of psychological distress among people receiving opioid agonist therapy.

We will also assess adherence to physical activity recommendations, physical functioning, symptoms of lung disease, assessment of changes in quality of life, fatigue, and inflammatory markers from a blood sample.

### Trial design {8}

A multicentre individually randomized controlled trial, parallel groups, 1:1 allocation ratio, superiority trial.

## Methods: participants, interventions, and outcomes

This protocol was developed from preliminary experiences from a multicentre pilot study evaluated with a mixed-method design (not yet published). In summary, the ATLAS4LAR study group conducted a 6-week pilot intervention with a group-based exercise program for people receiving OAT. This pilot study was conducted from May 2021 to June 2021. We aim to evaluate a prolonged scaled-up version of this intervention.

### Study setting and participants {9}

Recruitment will take place in OAT outpatient clinics in Bergen and Stavanger, two of the four largest cities in Norway. The Department of Addiction Medicine at Haukeland University Hospital in Bergen and OAT clinic in Stavanger have adopted an integrated treatment and care model for patients receiving OAT. In Bergen, OAT outpatient clinics are established in each district. Staff are multidisciplinary and include a consultant as well as a doctor specializing in addiction medicine, nurses, social workers, and psychologists. The patients are followed up almost daily with an observed intake of the OAT medications, such as methadone and buprenorphine treatment [22]. Bergen has suburb-based outpatient clinics while Stavanger has one main central outpatient clinic in addition to one for the neighboring areas. Except that, they are mostly similar. The treatment model in Bergen and Stavanger is a good platform to test the integration of additional interventions aiming to improve the health of a vulnerable group and gather knowledge, which traditionally has been very difficult to reach [23].

### Eligibility criteria {10}

The participants will be recruited from OAT outpatient clinics in Bergen and Stavanger inclusion criteria are as follows:

- Adults receiving OAT from the outpatient OAT clinics with weekly follow-ups.
- Giving informed consent.

Exclusion criteria:

- Not able to participate in the physical exercise intervention due to health disabilities.
- Being imprisoned or hospitalized.

### Who will take informed consent? {26a}

Research nurses from the included OAT clinics invited participants to take part in the study during an annual health assessment. Interested participants were asked to provide a written informed consent.

### Additional consent provisions for collection and use of participant data and biological specimens {26b}

Participants consented for participation, review of medical records, and for the collection of blood samples to assess biochemical indicators of inflammation.

## Interventions

### Explanation for choice of comparators {6b}

To reduce selection bias, participants in the intervention and comparison groups are recruited from the same OAT clinics. The comparison group follows standard treatment without offered exercise intervention. Norwegian standard care for OAT patients primarily focuses on opioid substitution with opioid agonist therapy. In contrast, our intervention arm extends this approach by testing the added value of physical activity and how this impacts on mental health.

### Intervention {11a}

The intervention is a supervised group-based exercise intervention for 16 weeks, which includes two outdoor sessions per week. The workout consists of two parts, the first focusing on endurance and the second focusing on resistance training. Every workout follows the same structure and lasts for approximately 45 min. The exercise starts with roughly 15 min of endurance warm-up, followed by moderate to high-intensity running or walking intervals. Eight repetitions of 30 s uphill, and the participants can freely choose between running and walking. The preferred intensity of the intervals is > 13 on Borg-score 20, meaning at least moderate-intensity activity [24]. The participants will be guided in using the Borg scale to identify exercise intensity, and the perceived exertion will be measured at the end of the endurance section. The resistance training consists of four exercises focusing on strengthening the large muscle groups, including pectoralis major, rectus abdominis, quadriceps femoris, gluteus maximus, and latissimus dorsi. The strength-training program follows the same structure as the intervals, 30 s of active time and approximately 60 s of break, four rounds.

The intervention is based on the World Health Organization 2020 guidelines on physical activity [25], and national guidelines for chronic obstructive pulmonary disorder [26]. In addition, a clinical therapist panel and users with extensive experience in exercise as therapy for drug and mental health disorders from Stavanger and Haukeland University Hospitals provided feedback on the intervention. We drew upon the preliminary experiences of patients and research personnel from the pilot study as well. Clinical staff, research staff, and people with user experience will supervise the exercise sessions. The supervisors have been trained to carry out this exercise program, and the research team will provide weekly guidance to these supervisors. Research nurses will perform random checks throughout the intervention period using the Borg-scale 20 [24], to determine whether intervention sessions use the desired intensity, or if any adjustments are needed.

### Criteria for discontinuing or modifying allocated interventions {11b}

If the participant asks to discontinue the allocated interventions, they are free to discontinue the intervention at any time.

### Strategies to improve adherence to interventions {11c}

The intervention will use existing infrastructure to improve adherence to the intervention. We will try to coordinate physical exercise sessions to link as best as possible with the clinical follow-up of the participants including the choice of timing of the session. Short text message reminders will also be sent to participants to ensure they remember the session.

### Relevant concomitant care permitted or prohibited during the trial {11d}

There are no restrictions on concomitant care during the trial.

### Provisions for post-trial care {30}

At their local OAT clinic, participants will receive yearly health assessments following the trial.

### Outcomes measures {12}

The data collection will take place within the patient’s local OAT clinic and the research nurse will lead the collection. The nurse has training in performing these tests, both as part of the annual assessment and in the pilot study.

Primary outcome measure:

Psychological distress

- The primary outcome is psychological distress 12–16 weeks after intervention initiation assessed with the Norwegian-validated translation of the ten-item version of the Hopkins Symptom Checklist (SCL-10) [27]. SCL-10 is a valid, reliable, relatively brief, and easy tool to implement for assessing psychological distress symptoms. SCL-10 will be evaluated with mean SCL-10 item score, and compared between the intervention and control arm.

Secondary outcome measures 12 to 16 weeks after intervention initiation:

- Physical functioning assessed with a 4-min step test measuring the number of steps climbed in the period [28].
- Physical activity assessed using the Norwegian-validated translation of the International Physical Activity Questionnaire (IPAQ) [29].
- Changes in fatigue will be assessed with the Fatigue Symptom Scale (FSS-3) [30].
- Changes in health-related quality of life assessed with EuroQoL EQ-5D-5L [31, 32] in addition to a self-reported question on happiness on a 0 to 10 scale.
- Changes in self-reported substance use will be assessed comparing change in overall substance use (using a semi-continuous substance use score from 0 to 1 including frequency of intake of several substances) by before intervention and in a 1-year follow-up visit [11].
- Adherence to OAT (as proportion in each arm who 1 year after initiation of the intervention is receiving OAT with active follow-up during last month).
- Biochemical indicators of inflammation (measured with C-reactive protein in serum and total leukocyte count in blood).
  - ◦ Sub-group (*n* = 60): IFN-gamma, IL-1beta, IL-1RA, IL-6, IL-8, IL-10, IL-17A, MCP-1, TNF-alfa measured in dried blood spots.

Baseline characteristics as predictors for degree of active participation in the physical activity interventions will be assessed.

### Participant timeline {13}

See Table 1: Flow chart of the study outlining follow-up visits and assessment at each visit.

**Table 1:** Flow chart of the study outlining follow-up visits and assessments at each visit

	**Screening (research nurse)**	**Treatment follow-up week 0 to 16 (nurses/social workers)**	**Intervention end-assessment (12–16 weeks after initiation)**	**Intervention post-assessment (10–30 weeks after intervention) initiation)**	**Annual follow-up**
**Research nurse assessment**	X		X	X	X
- Informed consent	X				
- Eligibility assessment	X				
- Follow-up by OAT staff		X			
- Clinical assessment	X		X	X	X
Biochemical tests	X		X	X	X
Physical funct. (4-min step test)	X		X	X	X
Physical activity. (IPAQ)	X		X	X	X
Psychological distress (SCL-10)	X		X	X	X
Fatigue symptoms (FSS-3)	X		X	X	X
Quality of life EQ-5D-5L	X		X		X

### Sample size {14}

Based on a cohort study in the same population [11], we assume that psychological distress assessed with a mean item score of SCL-10 score is 2.2 with a standard deviation of 0.8. To detect a reduction in psychological distress to a mean level of SCL-10 of 1.8 which is under the typically used threshold of 1.85 for substantial psychological distress [27], among participants taking active part in intervention and being evaluated among those randomized to intervention groups, with 62.5% of the participants randomized to the intervention assumed to take active part in intervention (i.e., a mean among those randomized to the intervention of 1.95), with 80% power a 1.1 intervention: control ratio, a two-sided test, and alpha (*α)* error of 5%. Based on these assumptions, 324 persons are required in total, including 162 persons in the intervention arm and 162 persons in the control arm (statistical power was calculated in Stata SE 17.0).

### Recruitment {15}

All patients receiving OAT from included OAT outpatient clinics will be considered the reference target population. A clinically based annual health assessment will be accessible to all patients in the included OAT clinics. Participants will be informed about the trial and invited to participate. Study personnel provides an extended evaluation for those giving informed consent and fulfilling the study eligibility criteria.

## Assignment of interventions: allocation

### Sequence generation {16a}

We will use 1:1 randomization that will be electronically registered using a randomization algorithm made through Stata that is linked to an electronic number for each patient (linked to CheckWare).

### Concealment mechanism {16b}

After all eligibility criteria have been met and consent has been obtained, a unique patient identifier number will be entered into a randomization spreadsheet to determine which study arms the participant will receive.

### Implementation {16c}

To enroll and assign participants, research nurses will use a randomization algorithm created through Stata linked to a patient's electronic number.

## Assignment of interventions: blinding

### Who will be blinded {17a}

Blinding of patients is regarded as complex and infeasible. Patients will be informed of the follow-up they will receive, but not of other follow-up alternatives that are used or the exact hypotheses for the study. Outcomes assessors and analysts will be blinded.

### Procedure for unblinding if needed {17b}

Not applicable. The research nurses know patient assignment.

## Data collection and management

### Plans for assessment and collection of outcomes {18a}

In both control and intervention groups, the outcome measures will be collected in the OAT clinics through a structured interview and clinical assessments of the participants.

**Fig. 1:**
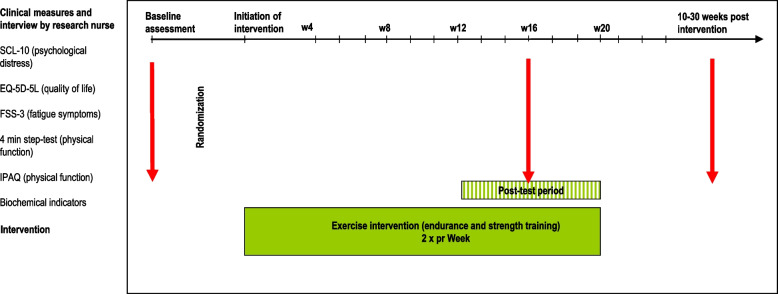
Illustrates the timing of the intervention and follow-up for the study. *Measurements are timed as indicated by the arrows

Research personnel with background as health professionals will perform the data collection. Data collection and follow-up will be given in line with Table 1 and Fig. 1.

### Plans to promote participant retention and complete follow-up {18b}

We assume participants will form a positive group dynamic which will promote participant retention. Further, we assume the intervention will be experienced as useful, which will also contribute to participant retention. Participants will also receive weekly appointment reminders and follow-ups from the research and clinical staff.

### Data management {19}

Research nurses will collect all data using electronic data collection software (Checkware®). This information will be stored on the University Hospital of Bergen’s secure servers. This study will collect clinical data from electronic medical records (DIPS®).

### Confidentiality {27}

All personal data is stored on a secure, access-restricted research server. The senior investigators LTF and JHV will import data from the collection software (Checkware®) and from the electronic medical record to a common file using each participant’s Norwegian personal identification number. Each participant is then given a computer-generated identification number for further analysis. Only anonymized data will be published.

Research nurses use paper forms for collecting the data during the trial and before data is entered into the collection software. Appointments are made using the medical record system. The research nurses store all paper forms that may be connected to a participant in a locked file in a room with restricted access.

For documentation and follow-up purposes, person-linkable data will be stored until the end of the project on the 31st of December 2029, and then deleted.

### Plans for collection, laboratory evaluation, and storage of biological specimens for genetic or molecular analysis in this trial/future use {33}

Blood samples with biochemical indicators of inflammation (C-reactive protein and leukocyte count with differentials) will be drawn from each participant at screening and at the intervention end-assessment.

## Statistical methods

### Statistical methods for primary and secondary outcomes {20a}

A detailed plan/protocol for analysis will be developed before data export and analysis adhering to the SPIRIT guidelines. Analysis methods and presentation of results will follow the CONSORT guidelines [33–35]. All tests will be two-sided. Descriptive results and efficacy estimates will be presented with 95% confidence intervals, and the statistical significance is set at *p* < 0.05. Potential confounders may be considered for adjustment if they are imbalanced at baseline, with assumed meaningful differences. Missing data will be considered, and appropriate imputations based on pre-defined assumptions will be done when necessary, as described in a detailed analysis plan. Categorical variables will be summarized as percentages and continuous variables as medians with interquartile ranges or means with standard deviation for variables with a Gaussian distribution. The primary outcomes will be analyzed with generalized linear models, Gaussian distribution. The main analyses will be analyzed as intention-to-treat.

### Interim analyses {21b}

There will be weekly meetings between the research nurses and the investigators throughout the study period. The meetings will evaluate the progression of the trial and adverse events.

### Methods for additional analyses (e.g., subgroup analyses) {20b}

We will also conduct per-protocol analyses, including those restricted to intervention participants actively taking part in the intervention for at least 8 of the 16 weeks (50%). This will be conducted for both primary and secondary outcomes. We will also conduct sub-group analyses for participants with less or more than the median number of steps in the 4-min step test.

### Methods in analysis to handle protocol non-adherence and any statistical methods to handle missing data {20c}

If any variables are missing of the primary outcome, they will be handled using an intention-to-treat strategy; the baseline value will be used.

### Plans to give access to the full protocol, participant-level data, and statistical code {31c}

Access to the complete protocol anonymized participant-level dataset and statistical code will be granted in parallel with publication.

## Oversight and monitoring

### Composition of the coordinating center and trial steering committee {5d}

The principal investigator, investigators, research nurses, and user representatives will meet once a week (study coordination unit). Research nurses and the primary investigator will meet weekly during the study period. The nurses and social workers providing weekly patient follow-up will meet daily with research nurses to ensure that both clinical aspects and research are well coordinated.

### Composition of the data monitoring committee, its role and reporting structure {21a}

There will not be an independent data monitoring committee, but the study coordination unit will ensure safety, adherence to the protocol, quality of the study, and ethical conduct.

### Adverse event reporting and harms {22}

All grade 3 (severe) and grade 4 (potentially life-threatening) adverse events are considered serious adverse events and will be reported to the study coordination unit that will ensure that proper follow-up will be provided, and if there are linked severe adverse events and significant differences between the arms, the trial will be terminated. Some might have adverse reactions to physical exercises and people with limited experience with exercise can find exercise unpleasant [36]. Participating in trials with an exercise-based intervention increases the risk of non-serious adverse events but not serious adverse events [37]. For safety evaluation, all serious adverse events occurring during the trial follow-up period will be recorded. According to current treatment guidelines, all serious adverse events will be followed until resolution or a stable clinical endpoint is reached. The research group led by the principal investigator, LTF will decide if it is necessary to terminate the trial.

### Frequency and plans for auditing trial conduct {23}

There are no plans for independent trial auditing. However, internal bi-annual audit procedures for study conduct and intervention will take place.

### Plans for communicating important protocol amendments to relevant parties (e.g., trial participants, ethical committees) {25}

Important protocol amendments will be submitted to the ethical committee.

### Dissemination plans {31a}

Outcomes of the trial will be published in peer-reviewed journals. We will also submit abstracts to relevant national and international congresses. Summaries of the outcomes will be provided to participants and clinical staff at the participating OAT clinics.

### Ethics {24}

In the study, participation is not assumed to pose a substantial risk. However, blood collection might be unpleasant, and exercise can be exhausting. The regional ethical committee has approved the study (no. 155386 REK sør/øst C, dated 23.09.2020/05.04.2022). The study is also registered online at ClinicalTrials.gov identifier: NCT05242848. The trial will be conducted according to the Declaration of Helsinki and other international conventions, GCP, and GLP standards [38, 39]. Each participant will be required to provide written informed consent and assent.

## Discussion

This multicentre randomized controlled trial aims to evaluate the effects of an integrated group-based physical exercise intervention on psychological distress in people with substance use disorders receiving OAT. To the best of our knowledge, no large randomized controlled trials with exercise interventions have been developed and tested among patients receiving OAT [15].

If successful, an exercise program could be integrated into treatment service. Integrated treatment models could be an alternative to increase adherence to OAT and lifestyle-related disorders. However, barriers to succeeding with exercise programs in clinical settings in this population include difficulties with adherence to the exercise program, difficulty devising a suitable intervention, and the relatively high cost of intervention related to personnel, equipment, or facilities [18, 40, 41].

Improving health seems to be one of the most important motives to start exercising for people with opioid use disorders. These findings are important factors to take into account when designing exercise interventions and using an already familiar setting to deliver treatment may lower the participation threshold to increase engagement [42].

Our trial has some limitations and several strengths. As mentioned earlier, complete blinding is hard to achieve. However, we aim to blind the outcome assessor and analyst in this study, and the data collectors have a strict protocol to follow when performing physical function tests [28]. All participants will receive the same instruction, reducing the possibility of external motivation from the data collector performing the 4-min step test. Additionally, since the study is individually randomized, the risk of confounding factors is minimized. The study population receiving OAT is large enough to answer the primary objectives accurately, and the associated accuracy for secondary objectives is assumed to be adequate.

If successful, this approach has the potential for widespread application through health care services for people in opioid agonist therapy.

## Trial status

Trial protocol version 4, 9^th^ of December 2024. Start of recruitment 19^th^ of April 2022. Estimated completion of recruitment December 2023.

## Data Availability

The final trial dataset will be made available to the authors mentioned under the “Acknowledgements” section.

## Abbreviations

OAT: Opioid agonist therapy
SCL-10: Hopkins Symptom Checklist
IPAQ: International Physical Activity Questionaire
FSS-3: Fatigue Symptom Scale
